# The Association between Daytime Napping and Cognitive Functioning in Chronic Fatigue Syndrome

**DOI:** 10.1371/journal.pone.0117136

**Published:** 2015-01-09

**Authors:** Zoe M. Gotts, Jason G. Ellis, Vincent Deary, Nicola Barclay, Julia L. Newton

**Affiliations:** 1 Faculty of Health and Life Sciences, Northumbria University, Newcastle-upon-Tyne, United Kingdom; 2 Institute of Cellular Medicine, Medical School, Newcastle University & Newcastle Hospitals NHS Foundation Trust and UK NIHR Biomedical Research Centre in Ageing, Newcastle-upon-Tyne, United Kingdom; Charité University Medicine Berlin, GERMANY

## Abstract

**Objectives:**

The precise relationship between sleep and physical and mental functioning in chronic fatigue syndrome (CFS) has not been examined directly, nor has the impact of daytime napping. This study aimed to examine self-reported sleep in patients with CFS and explore whether sleep quality and daytime napping, specific patient characteristics (gender, illness length) and levels of anxiety and depression, predicted daytime fatigue severity, levels of daytime sleepiness and cognitive functioning, all key dimensions of the illness experience.

**Methods:**

118 adults meeting the 1994 CDC case criteria for CFS completed a standardised sleep diary over 14 days. Momentary functional assessments of fatigue, sleepiness, cognition and mood were completed by patients as part of usual care. Levels of daytime functioning and disability were quantified using symptom assessment tools, measuring fatigue (Chalder Fatigue Scale), sleepiness (Epworth Sleepiness Scale), cognitive functioning (Trail Making Test, Cognitive Failures Questionnaire), and mood (Hospital Anxiety and Depression Scale).

**Results:**

Hierarchical Regressions demonstrated that a shorter time since diagnosis, higher depression and longer wake time after sleep onset predicted 23.4% of the variance in fatigue severity (p <.001). Being male, higher depression and more afternoon naps predicted 25.6% of the variance in objective cognitive dysfunction (p <.001). Higher anxiety and depression and morning napping predicted 32.2% of the variance in subjective cognitive dysfunction (p <.001). When patients were classified into groups of mild and moderate sleepiness, those with longer daytime naps, those who mainly napped in the afternoon, and those with higher levels of anxiety, were more likely to be in the moderately sleepy group.

**Conclusions:**

Napping, particularly in the afternoon is associated with poorer cognitive functioning and more daytime sleepiness in CFS. These findings have clinical implications for symptom management strategies.

## Introduction

Chronic Fatigue Syndrome (CFS) is a chronic condition characterised by intense fatigue of at least 6 months in duration and affects both physical and cognitive functioning. It is diagnosed by a) its duration, b) not being explained by another medical condition, and c) being associated with other symptoms such as myalgia, sleep disturbance and concentration difficulties [1]. So defined, CFS effects between 0.2–2.6% of the adult population and women are generally 2–3 times more affected than men [2]. Although the aetiology of CFS is largely unknown, it is likely to be multi-factorial with neuroendocrinological, autonomic, behavioural, cognitive and social factors interacting to provoke and/or maintain symptoms [3].

Whilst fatigue is the principal complaint, this is closely followed by sleep disturbance with 87–95% of CFS patients reporting unrefreshing sleep [4–7]. More specifically, patients tend to report fragmented sleep and sleep onset difficulties despite feeling tired [8], and one study found that 61% of patients reported sleep continuity complaints which were independent of cognitive functioning, fatigue and psychological wellbeing [9]. Disrupted sleep has been shown to cause fatigue, myalgia and poor concentration in healthy volunteers and therefore sleep disruption may be not just a consequence but also a cause of the other symptoms in patients with CFS [10].

‘Tired’ is a general term often derived from fatigue and sleepiness, which are used interchangeably. They are both related to unrefreshing sleep and show affective symptoms. It should be noted that fatigue and sleepiness are also common symptoms in patients with depression and in patients with sleep disorders. In particular, depressed patients show high sleepiness scores on the ESS [11] and patients with sleep disorders have markedly high scores on fatigue [12]. However, these two independent daytime symptoms remain poorly understood and are often blurred. Despite this, a clinical distinction between fatigue and sleepiness has been shown in patients with CFS and with a sleep disorder [13] but the distinction between these two phenomena remains difficult for both patients and clinicians. Given these symptoms have different implications in terms of diagnosis and treatment, there is an increased the risk for under diagnosed sleep disorders in fatigue patients [14, 15].

Daytime sleep, or napping, has been shown to have a negative impact on nocturnal sleep in non CFS populations. For example a long nap or a nap taken later in the day or early evening has been shown to have a detrimental effect on the length and quality of sleep during the subsequent night, by decreasing homeostatic pressure [16–19].

Disrupted sleep may serve to complicate the course of CFS by worsening existing symptoms. Even if sleep complaints are common in CFS, existing studies of self-rated sleep and its association with daytime functioning and disability are limited. Findings show sleep complaints are associated with greater global disability and that sleep continuity complaints become worse when the underlying condition and fatigue worsen [20]. Further, identifying sleep disturbances to form a key dimension of CFS (alongside others such as social functioning, psychological wellbeing and functional impairment) [9], also that CFS patients report more naps and restless legs than healthy controls [20]. However, studies have not directly examined self-reported sleep and its impact on daytime physical and mental functioning in CFS, nor have they looked at the impact of daytime napping in this population. Importantly the methodological limitations (i.e. taking measures of sleep disturbance based on self-rated presence or absence of sleep problems [20], not defining the duration of sleep complaints [9], having a limited range of complaints [9, 21] or examining sleep related complaints lasting only one night [21]) are problematic and do not meet the standard for sleep assessment (14-day consecutive sleep diaries) to afford appropriate characterisation of patient’s sleep patterns. It is also reasonable to consider psychological factors as potential contributors to daytime functioning in CFS, given the well-established relationship between anxiety, depression and fatigue [22]. Likewise, consideration of illness length and gender is important in CFS research, given the variable nature of the disease and a condition that primarily affects women [23]. The present study therefore aims to examine self-reported sleep in men and women with CFS, and to establish whether disrupted sleep continuity and daytime napping predict markers of daytime functioning, specifically fatigue severity, sleepiness and cognitive dysfunction. The present study also aims to investigate potential associations between illness length, anxiety, depression and daytime functioning.

## Methods

### Ethics Statement

The study was approved by the Newcastle and North Tyneside Local Research Ethics Committee and all subjects provided written informed consent.

### Subjects

Based on a similar study [24], a power calculation (significance level of 0.05 and statistical power of 80%) was performed to estimate participant numbers for this study. In order to detect the smallest change of clinical relevance, a sample size of 87 subjects was required. One hundred and eighteen adults (allowing for a 10% drop out rate) were recruited from a specialist CFS service and met the 1994 CDC diagnostic criteria for CFS [1]. Participants had been screened by a specialist physician for any medical and mental illness which could explain their fatigue, as per the UK NICE (British National Institute for Health and Clinical Excellence) Guidelines for CFS [25]. Participants had completed sleep diaries and functional assessments of fatigue, sleepiness, cognition and mood as part of their usual care for CFS.

### Assessment

**Subjective sleep assessment.** Subjective sleep was assessed using a 14-day sleep diary. The diary was based on a sleep diary that has been used previously and demonstrated validity for research use in CFS populations, allowing participants to monitor their sleep experience on a daily basis [24]. Patients were required to complete the diary on waking each morning. Patients recorded the times at which they retired to bed, identified time of lights out, the number of nocturnal awakenings, time of morning awakening, the number, duration and timing of daytime naps, alcohol and caffeine consumption, and medication use. Patients returned the completed diaries and data were averaged across the number of days completed (Mean completion 14 ± 0 days). The following sleep continuity variables were calculated; Time in Bed (TIB), Total Sleep Time (TST), Sleep Onset Latency (SOL), Wake After Sleep Onset (WASO), Number of Awakenings (NWAK), and Sleep Efficiency (SE). To characterise patient’s napping behaviour, an overall duration of daytime napping was calculated in minutes, including duration of napping that occurred in the morning (AM napping) and afternoon-evening (PM napping) period, on average across the 14 days. (Descriptions of sleep variables are detailed in table 1).

**Table 1:** Description of sleep variables.

**Abbreviated Variable**	**Sleep Variable (measure)**	**Description**
**TST**	Total Sleep Time (minutes)	Amount of time asleep
**SOL**	Sleep Onset Latency (minutes)	Length of time from lights out to first episode of stage 2 sleep
**WASO**	Wake After Sleep Onset (minutes)	Number of minutes of recorded wake following first episode of stage 2 sleep
**NWAK**	Number of Awakenings (over TSP)	Number of wake bouts following first episode of stage 2 sleep
**SE**	Sleep Efficiency (%)	Percentage of time spent asleep from the amount of time spent in bed (TST/TIB*100)

Notes: REM, rapid eye movement; TSP, total sleep period; TST, total sleep time

**Functional and symptom assessment tools**. Daytime functional assessments were completed by patients as part of their usual care. Levels of daytime sleepiness were determined by the Epworth Sleepiness Scale (ESS). Patients self-report their chance of dozing or sleeping based on 8 given situations. The responses are made on a Likert-type scale ranging from 0 (would *never* doze/sleep) to 3 (*high* chance of dozing/sleeping) Total possible scores derived from the 8 questions range from 0–24, with a score of 10 or more being indicative of significant daytime sleepiness [26].

The 11-item Chalder Fatigue Questionnaire (CFQ) evaluated fatigue severity. It is one of the most widely used measures for assessing physical and mental symptomatic fatigue experienced by CFS/ME patients. Four response options are available, ranging from ‘‘less than usual’’ to ‘‘much more than usual’ The Likert system for scoring was used (0, 1, 2, 3), with a total possible score ranging from 0–33. A higher score indicates more fatigue. The test has been shown by its authors to have good reliability (r =.86 for physical fatigue, and r =.85 for mental fatigue) and has high internal consistency as measured by Cronbach’s alpha (.89) [27].

The Trail Making Test (TMT) was used to assess the cognitive domain of executive functioning and speed of information processing. The task requires participants to connect-the-dots between 25 consecutive targets on a sheet of paper. In version A of the test all of the targets are numbers. In version B, targets alternate between numbers and letters (1, A, 2, B). The time it takes to complete the task is the measure of performance, with longer time indicating poorer cognitive performance [28]. The TMT has been shown to demonstrate neurocognitive impairments in CFS populations, with reduced performance on speed of information processing compared to healthy controls [29, 30]

Patients completed the 25-item Cognitive Failures Questionnaire (CFQ), a tool developed by Broadbent [31] to assess self-reported deficits in attention, perception, memory and motor functioning. The questionnaire measures the frequency of everyday cognitive failures or lapses by asking participants to rate how often they make mistakes on a 5-point Likert scale, from 0 (never) to 4 (very often). The instrument produces a global “cognitive complaints” score (ranging from 0–100), with higher scores indicating more cognitive failures. When compared with healthy controls, individuals with CFS have shown higher levels of global “cognitive complaints” on the CFQ [32–34].

To control for the impact of anxiety and depression on daytime functioning, patients completed the 14-item Hospital Anxiety and Depression Scale (HADS). Items were rated on a four-point Likert scale (0–3); seven items provide a measure of anxiety and seven a measure of depression, with scores ranging from 0–21 on each subscale [35]. For both Anxiety and Depression scales, raw scores between 8 and 10 identify mild cases, 11–15 moderate cases and 16 or above, severe cases.

### Statistical analysis

The data derived from the 14 days sleep diaries and momentary functional assessments of fatigue, sleepiness, cognition and mood, were pooled for each patient prior to analysis. The data were analysed with SPSS (IBM 19.0). First, descriptive statistics for CFS patients’ self-reported sleep variables and napping were calculated. HADS variables were also compared with published data of a reference sample [36]. Second, Multiple Regression analyses examined the extent to which self-reported sleep and napping predicted patient’s daytime physical and mental functioning, fatigue severity and sleepiness. Step one of each model incorporated patient characteristics (age, gender, length of disease), step two consisted of the mood variables of anxiety and depression (from HADS), and step three contained the subjective sleep parameters (SE, NWAK, WASO, SOL, napping duration), TIB and TST were not included in the models as together these variables make up SE. Daytime symptoms measured by fatigue severity (Chalder Fatigue Scale), daytime sleepiness (ESS) and objective and subjective cognitive functioning (Trail Making Test, Cognitive Failures Questionnaire) were included as dependent variables in each analysis, respectively, and checks for multicollinearity were carried out. Casewise deletion due to missing data was defined on the basis of; a) 5% or more of an overall scale missing and/or b) less than 14 continuous days of sleep diary recorded.

## Results

Of the 118 sleep diaries returned by patients, 17 had missing data, and so 101 were used in the analysis. The final sample therefore consisted of 101 patients with an average length since diagnosis of 7.8 years (±7.34). The mean age of the sample was 42.05 (±12.99), range 16–68 years, and 81.2% were female.

21 (20.8%) patients in the sample did not report any daytime napping throughout the 14-day period. Of the 80 (79.2%) that did nap, on average napping duration was 39.55 (±55.35) minutes. Patients spent on average 7.80 (±16.03) minutes napping in the morning period (AM), and 31.74 (±43.94) minutes napping in the afternoon-evening period (PM). (The mean values for all sleep and functioning variables of the study, including demographic characteristics are shown in Table 2).

**Table 2:** Self-reported sleep, functional & symptom assessment data for CFS patients.

	***N***	**Mean (SD)**
**Patient Characteristics**		
Gender M, F [N (%)]	101	M: 19 (19%), F: 82 (81%)
Age	101	42.05 (12.99)
Length of Illness [Months]	101	93.69 (88.12)
**Sleep Continuity Parameters**		
Sleep Onset Latency (SOL) [Minutes]	101	37.60 (41.67)
Time In Bed (TIB) Sleep Period [Minutes]	101	569.50 (83.18)
Total Sleep Time (TST) Sleep Period [Minutes]	101	426.69 (80.30)
Number of Awakenings (NWAK)	101	1.48 (1.14)
Wake After Sleep Onset (WASO)[Minutes]	101	50.81 (43.75)
Sleep Efficiency (%)	101	75.74 (13.64)
**Daytime Sleep (napping)**		
Total Napping Duration [Minutes]	80	39.55 (55.35)
Duration AM Napping [Minutes]	80	7.80 (16.03)
Duration PM Napping [Minutes]	80	31.74 (43.94)
**Functional and Symptom Measures**		
HADS Anxiety	101	8.31 (4.62)
HADS Depression	101	8.42 (4.10)
Chalder Fatigue Questionnaire	101	25.19 (6.31)
Epworth Sleepiness Scale	101	9.87 (5.07)
TRAIL Making Test [Minutes]	96	135.31 (74.03)
Cognitive Failures Questionnaire	89	58.45 (21.14)

Notes: *N* differs on different measures. Data *presented as means and standard deviations (SD)*. HADS, hospital anxiety and depression scale

The sleep diary data show patients had on average TST of 426.69 (±80.30) minutes, SOL of 37.60 (±41.67) minutes, WASO of 50.81 (±43.75) minutes and sleep efficiency at 75.74 (±13.64)%, demonstrating values in the abnormal range [37].

### Fatigue severity

For fatigue severity (based on Chalder Fatigue Questionnaire scores), three predictors (length of disease, depression, WASO) accounted for 24% of the variance. Examination of the beta weights in the third model showed that length of disease significantly predicted Chalder Fatigue scores, with people who had been diagnosed for less time being more fatigued. Depression scores and WASO were the other significant factors (see Table 3).

**Table 3:** Hierarchical regressions for the dependent variables; Chalder Fatigue Scale, Cognitive Failures Questionnaire, Trail Making Task and Epworth Sleepiness Scale.

	**Dependent Variable**							
	**Chalder Fatigue Scale (*N = 101*)**		**Epworth Sleepiness Scale *(N = 101*)**		**Cognitive Failures Questionnaire (*N = 89*)**		**Trail Making Task (*N = 96*)**	
*Variables in the model*	β	*t*	β	*t*	β	*t*	β	*t*
Step 1								
Constant		10.11		3.54		5.07		5.03
Length of Disease	-0.41**	-3.27	0.00	0.01	0.13	1.17	-0.03	-0.28
Age	0.23*	2.22	0.15	1.36	0.08	0.75	0.11	0.99
Gender	-0.09	-0.96	0.03	0.33	0.09	0.82	-0.24*	-2.36
***Adj*.** **R^2^**	0.08		-0.01		0.00		0.03	
**F**	4.05**		0.75		1.05		2.12	
Step 2								
Constant		8.04		1.68		2.90		3.32
Length of Disease	-0.31**	-3.20	0.00	0.03	0.12	1.30	-0.01	-0.05
Age	0.11	1.06	0.10	0.93	-0.08	-0.84	0.01	0.10
Gender	-0.00	-0.04	0.09	0.89	0.24*	2.48	-0.17	-1.67
Anxiety	-0.02	-0.17	0.27*	2.41	0.33**	3.10	0.04	0.31
Depression	0.42***	3.77	0.11	2.41	0.34**	3.02	0.33**	2.70
***Adj*.** **R^2^**	0.22		0.09	0.96	0.30		0.13	
**F**	6.61***		3.05*		8.41***		3.73**	
Step 3								
Constant		3.38		-0.79		2.40		2.24
Length of Disease	-0.41***	-3.94	0.07	0.59	0.10	0.96	-0.02	-0.20
Age	0.07	0.62	0.11	1.03	-0.12	-1.17	-0.03	-0.31
Gender	-0.01	-0.09	0.11	1.16	0.23*	2.37	-0.16	-1.64
Anxiety	-0.08	-0.68	0.29*	2.48	0.32**	2.86	-0.02	-0.20
Depression	0.45***	4.02	0.12	1.04	0.30*	2.55	0.35**	2.81
SE (%)	0.05	0.39	0.20	1.66	-0.17	-1.43	-0.10	-0.80
NWAK	-0.18	-1.52	0.07	0.54	0.11	0.88	-0.11	-0.85
WASO (min)	0.34**	2.47	-0.09	-0.58	-0.02	-0.11	0.01	0.08
SOL (min)	-0.03	-0.28	0.02	0.18	0.01	0.08	-0.05	-0.46
Total Napping (min)	-0.05	-0.55	0.17	1.76	0.06	0.62	0.22*	2.19
***Adj*.** **R^2^**	0.24		0.14		0.30		0.14	
**F**	4.10***		2.57**		4.76***		2.49*	

Notes:

*p < .05,

** *p* < .01,

*** *p* < .001;

Entries represent standardized beta coefficients.

SE, sleep efficiency; NWAK, number of awakenings; WASO, wake after sleep onset; SOL, sleep onset latency

### Daytime sleepiness

Anxiety was the single determinant of daytime sleepiness and explained 14% of the variance in scores on the Epworth Sleepiness Scale. Higher anxiety significantly predicted higher scores on the ESS, based on the third model (see Table 3).

### Cognitive functioning

With regards to subjective cognitive functioning, three predictors (gender, anxiety and depression) explained 30% of the variance in scores on the Cognitive Failures Questionnaire (CFQ). Examination of the beta weights in the third model showed that gender significantly predicted subjective cognitive dysfunction, with women reporting more cognitive failures than men. Higher scores on anxiety and depression were also significant predictors (see table 3).

Patients’ Trail Making Test performance (objective cognitive functioning) was within the normal range for both TMT part A (mean = 43.79 ± 30.02 seconds) and part B (mean 92.83 ± 49.85 seconds) (completion time >78 seconds (TMT A) and > 273 seconds (TMT B) are indicative of cognitive impairment [20]). For the regression, Total Trail time was used. Two predictors (depression scores and napping duration) explained 14% of the variance on TMT performance, based on the third model that incorporated the self-reported sleep variables. Examination of the beta weights in the third model showed that having a higher depression score and a longer duration of *overall* daytime napping significantly predicted poorer TMT performance (longer completion time on the test) (see Table 3).

To explore the specific time of day at which napping occurred and determine whether this made a difference to performance on the TMT task, a further Multiple Regression analysis was carried out. Step three of the model which incorporated the subjective sleep parameters was modified to include total AM (morning) napping duration and total PM (afternoon-evening) napping duration, in place of total napping duration. Three predictors (depression scores, AM napping duration and PM napping duration) explained 19% of the variance in time taken to complete the TMT. Examination of the beta weights in this revised third model indicated that having a higher depression score, a longer duration of afternoon napping (β = .49) and less morning napping (β = -.30) were determinates of poorer objective cognitive functioning (see table 4).

**Table 4:** Hierarchical regression for the dependent variable; Trail Making Task (replacing total napping duration with AM and PM napping duration in the model).

	**Trail Making Task**	
*Variables in the model*	β	*t*
Step 1		
Constant		5.03
Length of Disease	-0.03	-0.28
Age	0.11	0.99
Gender	-0.24*	-2.36
***Adj*.** **R^2^**	0.03	
**F**	2.12	
Step 2		
Constant		3.32
Length of Disease	-0.01	-0.05
Age	0.01	0.10
Gender	-0.17	-1.67
Anxiety	0.04	0.31
Depression	0.33**	2.70
***Adj*.** **R^2^**	0.13	
**F**	3.73**	
Step 3		
Constant		2.30
Length of Disease	-0.02	-0.17
Age	-0.06	-0.54
Gender	-0.14	-1.39
Anxiety	0.00	0.03
Depression	0.29*	2.36
SE (%)	-0.11	-0.89
NWAK	-0.13	-1.01
WASO (min)	0.13	0.81
SOL (min)	-0.07	-0.63
Napping AM (min)	-0.30*	-2.15
Napping PM (min)	0.49**	3.41
***Adj*.** **R^2^**	0.19	
**F**	3.03**	

Notes: *N* = 96

* *p* < .05,

** *p* < .01,

*** *p* < .001;

Entries represent standardized beta coefficients

SE, sleep efficiency; NWAK, number of awakenings; WASO, wake after sleep onset; SOL, sleep onset latency

## Discussion

This study sought to characterize the sleep and daytime napping of patients presenting with CFS and the extent to which their self-reported sleep and napping behaviour impacted upon the daytime symptoms. The major findings of this study are: (i) CFS patient’s self-reported WASO, SOL and Sleep Efficiency are in the abnormal range, but also highly variable, (ii) higher self-reported depression, more WASO and a shorter time since diagnosis together explained 24% of patient variance in fatigue severity on the Chalder Fatigue Scale, (iii) scoring more highly on depression and longer duration of PM napping, with shorter amounts of AM napping explained 14% of variance on objectively assessed cognitive functioning (TMT). Being female and scoring more highly on anxiety and depression predicted 30% of the variance in self-reported daily cognitive failures (CFQ); (iv) Patients with higher scores of self-rated sleepiness (ESS) were characterised by those who self-reported higher levels of anxiety on the HADS, explaining 14% of the variance on the ESS.

### The role of sleep in daytime functioning

Recent work [38] would suggest that there are distinct, objectively verifiable, sleep phenotypes in the CFS population, and the variability in the present population may be reflecting this for subjective variables also. For instance, the mean sleep onset latency was 37.6 minutes but the standard deviation was 41.67 minutes. As such, whilst the present results are suggestive of the role of disturbed sleep in the CFS population as a whole, future work should be mindful that the nature of CFS patients’ sleep problems may differ but may fit the characteristics for a sleep-specific phenotype.

Given this caveat, sleep still emerges as a significant predictor of impaired daytime functioning. Disturbed sleep at night, specifically longer amounts of wake time during the sleep period, is significantly associated with daytime fatigue, and longer duration of napping during the afternoon-evening period is associated with objective measures of daytime cognitive impairment. This would suggest that rather than being “primary symptoms” of CFS, daytime fatigue and cognitive dysfunction may in part be mediated by disturbed sleep and daytime napping. This in turn would suggest that interventions, such as the sleep management strategies that form part of cognitive behavioural therapy for CFS, may impact on these symptoms by way of improving sleep. Daytime napping is a common target of CFS management interventions. In particular, Cognitive Behavioural Therapy for Insomnia (CBT-I) discourages napping during the course of treatment; excessive napping and extended time in bed are considered factors that can amplify existing disturbances in night-time sleep by weakening the homeostatic sleep drive [39]. The present study would add that daytime napping impairs cognitive function and may lead to a vicious circle where such napping causes daytime sleepiness which in turn leads to daytime napping.

### The role of other factors

Overall this study suggests that sleep is only one of the factors associated with daytime functioning in CFS as WASO was the only sleep parameter to influence fatigue. The other key predictors were scoring higher on the depression scale of the HADS and having a more recent diagnosis. The latter is interesting in that it would suggest that there is an “acute” phase to CFS, whereby fatigue is higher. This would fit with the qualitative study described in the previous chapter, and reports of patients in clinic, who frequently mention having learned to adjust to the disease and to pace themselves the longer they have it. This has particular implications for early stage treatment strategies, which should be involve helping people adjust to and manage their condition. The depression finding is less easy to interpret. Patients are rightly wary of being diagnosed as depressed and the overall means of this group are at the very low end of caseness. Overall the most parsimonious interpretation of these results would be that fatigue is influenced by multiple factors, and that this study has highlighted that sleep, adjustment to illness, and mood may be pieces in the complex biopsychosocial fatigue jigsaw. Again this would also suggest that helping people to adjust and adapt to illness, sleep management strategies, and mood management may impact positively on daytime fatigue.

Scores on the depression scale, and scores on the anxiety scale, also emerged, along with daytime napping, as significant contributors to objective and subjective measures of cognitive functioning. Anxiety also emerged as a single predictor of levels of daytime sleepiness. Any explanation of this is speculative, but it could be that higher anxiety, marked by higher autonomic arousal, may produce more sleepiness.

Overall these results suggest the determinates of daytime fatigue severity, sleepiness and cognitive functioning in CFS are multi-factorial. Whilst sleep is an important determinant, particularly disturbed sleep and daytime napping, other factors are also important. As such, any intervention probably needs to consider each factor on an individual basis. Whilst one person may benefit from straight forward sleep management, another person relatively early post-diagnosis may also need help adjusting to the illness, whilst others still may need help with the emotional impact of being ill. As there is no one sleep phenotype, there is no one typical CFS patient. It is therefore recommended that at the minimum patients receive an individualised sleep assessment by an experienced clinician and a sleep and napping diary.

There are several limitations to this study. Given that it is not standard procedure for patients presenting with fatigue in primary care to undergo routine sleep tests in a laboratory, they are at increased risk of having an undiagnosed sleep disorder and potentially being misdiagnosed and incorrectly treated. Given the occurrence of sleepiness and sleep disturbances in this sample, further sleep investigation is warranted, to exclude potentially undiagnosed sleep disorders such as PLMS and Apnea, which if treated, may also alleviate fatigue. The main methodological problems were that we largely relied on self-report data. Further, the time of day at which functional measures are taken should be considered for future studies, particularly where daytime napping occurs, as sleep inertia following a nap may have implications for cognitive performance [40]. As for objective measures, there was no clinical screening, by way of standardised interview for sleep disorders, no multiple sleep latency testing (MSLT) to objectively measure daytime sleepiness, and no polysomnography (PSG) to objectively measure sleep parameters. Nevertheless, subjective reports are a good way to identify the parameters of interest for future studies, and they are also what are routinely used in the clinic with CFS patients to assess treatment outcomes. Future work should consider a triangulation of subjective and objective reports of sleep, fatigue, sleepiness, and daytime functioning, both in the lab and in intervention studies. In terms of the latter, the present works suggests that sleep interventions merit further study in this population.

In conclusion, disturbances in sleep continuity may serve as a mediator of daytime mental and physical dysfunction in CFS. Whilst they need to be considered in the context of other factors, it would seem that targeting disturbed sleep and napping may improve daytime fatigue and cognitive functioning. Most current interventions in CFS are multi-factorial, and so involve sleep management strategies. However to date there has been no trial of sleep interventions on their own. The present study would suggest that this is an avenue worth exploring.
